# Enhanced distance perception in automated robotic endoscopy through scale-aware monocular depth estimation

**DOI:** 10.1007/s11548-026-03591-6

**Published:** 2026-04-14

**Authors:** Ruofeng Wei, Kai Chen, Yiyao Ma, Bin Li, Yunhui Liu, Qi Dou

**Affiliations:** 1 Department of Computer Science and Engineering, The Chinese University of Hong Kong, Shatin, Hong Kong China; 2 Department of Mechanical and Automation Engineering, The Chinese University of Hong Kong, Shatin, Hong Kong China

**Keywords:** Distance perception, Scale-aware monocular depth estimation, Geometric modeling, Endoscopic robotic surgery

## Abstract

**Purpose:**

The increasing demand for distance perception in surgical robotic systems is crucial for ensuring safe and effective operations in minimally invasive procedures. Accurate distance awareness allows surgical robots to autonomously navigate complex anatomical structures, particularly in endoscopic environments with limited visual feedback. Existing distance estimation methods often rely on expensive sensors that can pose safety risks and are ineffective under these conditions. To address this challenge, this work develops a monocular image-based framework leveraging geometric modeling and long-term tracking technologies to enable accurate distance measurements from a monocular camera, enhancing current endoscopic practices.

**Methods:**

The proposed approach introduces an endoscopic image-based pipeline designed to estimate high-quality, scale-aware depth from monocular endoscopic scenes. This pipeline utilizes surgical instruments with cylindrical shafts of known radius as geometric constraints to recover absolute scale. An automatic module is designed for continuous tracking of specific tissue regions and instruments within the scene, allowing for precise distance calculations between various objects.

**Results:**

Compared to state-of-the-art baselines, the depth model reduces mean absolute error from 8.56 mm to 6.35 mm and improves accuracy from 76.9% to 88.0%, while achieving near-perfect absolute scale estimation. The overall framework attains average distance errors between 4.7 and 6.4 mm across multiple measurement types, demonstrating reliable and efficient performance in complex endoscopic scenarios.

**Conclusion:**

We present a novel image-based distance perception framework aimed at enhancing surgical robot automation. The framework facilitates efficient measurements of various objects within endoscopic views. Through multiple distance-augmented endoscopic videos, we demonstrate the potential of our work to benefit robotic surgery tasks, including automatic navigation.

## Introduction

Robotic surgery has revolutionized minimally invasive procedures, providing unprecedented precision, dexterity, and control [1]. As the field advances toward automated robotic surgery [2, 3], the ability of surgical robots to autonomously perceive and interpret their environment becomes increasingly critical [4, 5]. A key aspect of this perception is distance awareness, the capacity to accurately assess spatial relationships within the surgical field. This capability is vital for ensuring safe and effective operations, especially in endoscopic procedures where robots must navigate complex anatomical structures [6] with limited visual feedback. Traditionally, distance estimation methods rely heavily on advanced sensors [7]. However, these sensors can be expensive, and their effective use is often limited by specific environmental conditions [8, 9]. Additionally, the integration of heavy sensors into surgical robots may pose safety risks, making them unsuitable for critical scenarios like minimally invasive surgery [10]. Moreover, for most surgical robots, a camera is the only embedded sensor. Therefore, a robust image-based perception framework is essential to calculate the distance.

### Problem formulation

Distance perception in endoscopic surgery relies on depth estimation from monocular images, which is fundamentally ill-posed due to the loss of absolute scale in 2D projections. This scale ambiguity prevents distinguishing between small nearby and large distant objects without extra constraints. The challenge is compounded by factors unique to endoscopy, including deformable tissues that violate static scene assumptions, specular reflections causing misleading depth cues, narrow fields of view limiting context, and uniform tissue textures that reduce visual features. Existing methods like stereo vision are unsuitable for predominantly monocular flexible endoscopes; SLAM and visual odometry struggle with non-rigid tissue deformations, and deep learning approaches fail to resolve scale ambiguity without ground-truth scaling and often sacrifice detail due to low-resolution inputs needed for real-time performance on embedded platforms.

In recent work [11], we addressed these challenges using a geometric constraint: the 3D modeling of surgical instruments with cylindrical shafts from images. Unlike methods that introduce additional sensors [12], this approach used the known dimensions of these instruments to recover the absolute scale of the scene, thus improving the accuracy of scale-aware depth estimation from monocular endoscopic images. Furthermore, combining depth estimates obtained at multiple resolutions was explored to enhance the quality of monocular depth, without requiring complex network architectures [13] or auxiliary tasks [14, 15]. However, scale-aware depth estimation alone is insufficient to achieve complete distance awareness in automated robotic endoscopy. To accurately measure the distance between surgical instruments and target tissues or between specific regions of the scene and the endoscope, continuous localization of these regions or parts of the instrument is necessary. While depth estimation establishes the foundational 3D geometry, the localization of objects and regions of interest is equally critical for precise distance computation.

### Contribution

To address this gap, we present the first comprehensive image-based framework for surgical robot distance perception, incorporating scale-aware depth estimation from monocular endoscopy. The proposed image-based pipeline estimates the scale-aware depth of monocular endoscopic scenes, by utilizing surgical instruments with cylindrical shafts of known radius as geometric constraints to recover absolute scale. We also propose a module for the continuous localization of specific tissue regions and instrument parts within the scene. By integrating these two modules, accurate distance calculation between various objects is achieved. The main contributions are summarized as follows:

- A comprehensive endoscopic image-based framework to enable surgical robots with distance perception.
- A real-time localization technique to track specific tissue regions and instrument parts within endoscopic views.
- Application of this image-based distance calculation framework to augment current endoscopy, significantly aiding future automated robotic surgeries.

This work extends a preliminary version presented at MICCAI 2024 [11] with major improvements:

- Integration of a real-time localization technique into the previous scale-aware depth estimation pipeline to achieve distance perception for surgical robots.
- Development of a localization technique tailored for the dynamic and complex environments of endoscopic surgery.
- Demonstration of multiple distance-augmented endoscopic videos using our image-based framework.

The remainder of this paper is organized as follows. Sect.  2 introduces the proposed framework systematically. Sect.  3 describes the experimental procedures. Sect.  4 evaluates the proposed method through experiments based on various datasets. Key issues and future works are discussed in Sect. 5, followed by conclusions in Sect. 6.

## Method

**Fig. 1:**
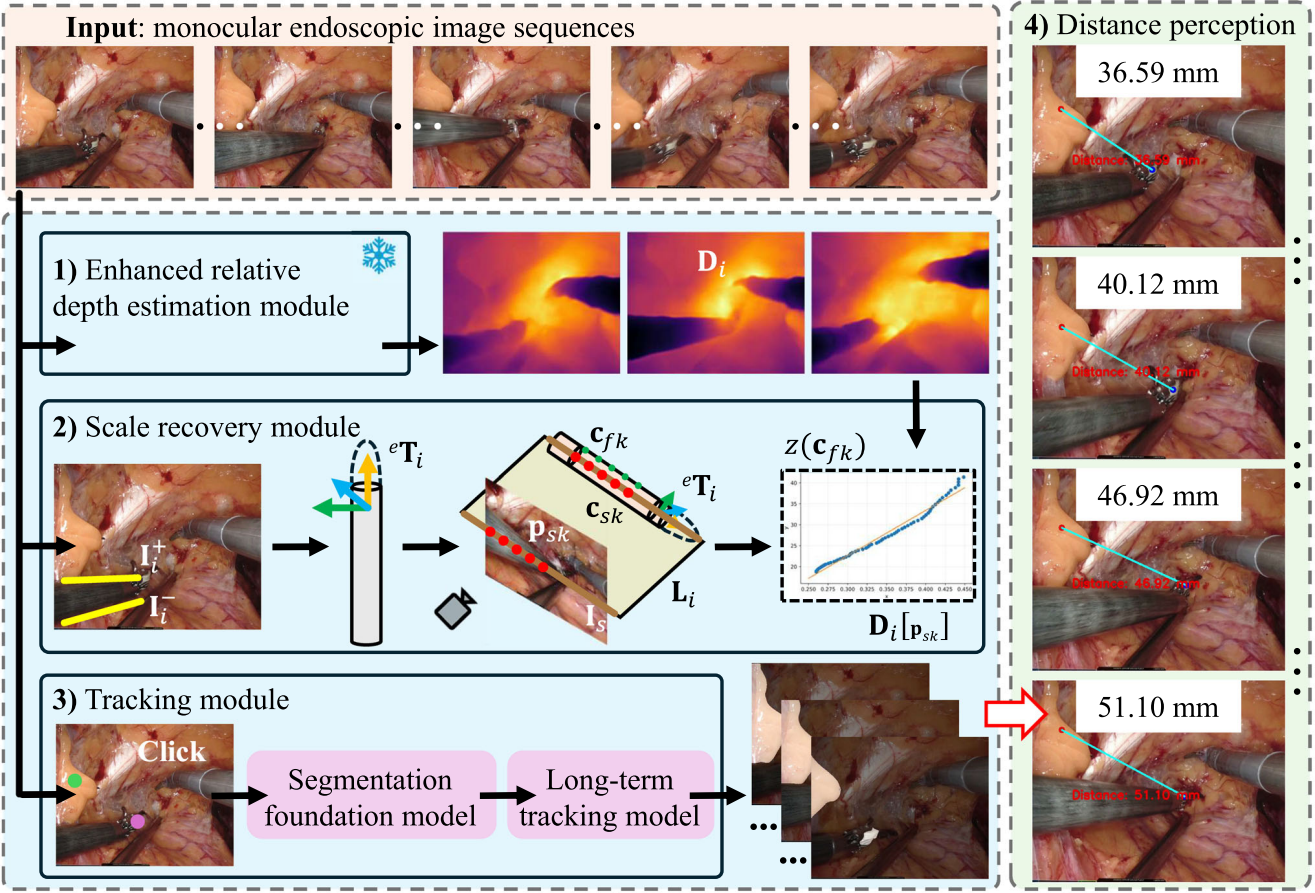
Overview of the proposed image-based distance perception framework for robotic endoscopy. Our framework takes the monocular endoscopic image sequences as **input**. The framework consists of three modules: **1)** Enhanced relative depth estimation module, **2)** Scale recovery module, and **3)** Tracking module. By integrating these modules, the proposed framework equips surgical robotic systems with **4)** distance perception awareness

Figure 1 presents an overview of the proposed scale-aware distance perception framework, which consists of three key modules. First, we utilize a relative depth estimation network to generate high-quality depth maps by fusing estimations from different image resolutions. Second, a geometric modeling method is introduced to calculate the 3D poses of surgical instruments within the scene, using only geometric primitives extracted from endoscopic images. By integrating the instrument pose with the depth map, we can recover the scale between relative depth and real-world values, enabling estimation of absolute depth in monocular endoscopic scenes based on geometry of surgical instruments. Finally, an automatic tracking module is developed to localize specific tissue regions and parts of surgical instruments in real time, which is integrated into the distance perception system. Detailed descriptions of each module follow.

### Enhancing depth estimation with multi-resolution fusion

Our approach to relative depth estimation is grounded in the Monodepth2 framework [16]. During training, the network is optimized by minimizing the photometric loss [17] between actual input images and synthetic frames generated through novel view synthesis. However, cropping input images to lower resolutions during training results in a significant loss of detail, which negatively impacts the accuracy of depth estimation. To tackle this challenge, an enhanced module has been developed to merge two depth estimates of the same endoscopic image obtained at different resolutions, thereby improving the overall quality of relative depth maps. This depth estimation model is designed to accommodate images of varying sizes. When images at different resolutions are fed into the network, we notice distinct patterns in the resulting depth maps. At lower resolutions, which closely match the training data, the depth estimations effectively represent the overall structure but often miss finer, high-frequency details. In contrast, higher-resolution inputs capture more intricate details, but may suffer from reduced structural consistency. Notably, by fusing depth estimates from different resolutions, detailed information from the higher-resolution input is transferred to the lower-resolution depth map while preserving its structural integrity. To improve the lower-resolution depth maps, we introduce a specialized enhanced module that produces two depth estimates for the same endoscopic image: one derived from a lower-resolution input, which maintains structural consistency but lacks detailed features, and another from a higher-resolution input, which is rich in detail. A low-pass fusion filter [18] is applied, using the low-resolution depth map as a guide to refine the high-resolution estimate. This fusion process results in a relative depth map that effectively balances structural integrity with detailed information. As a result, we can accurately predict the enhanced relative depth map $\textbf{D}_i$ for each monocular image.

### Scale recovery via surgical instrument pose estimation

**Fig. 2:**
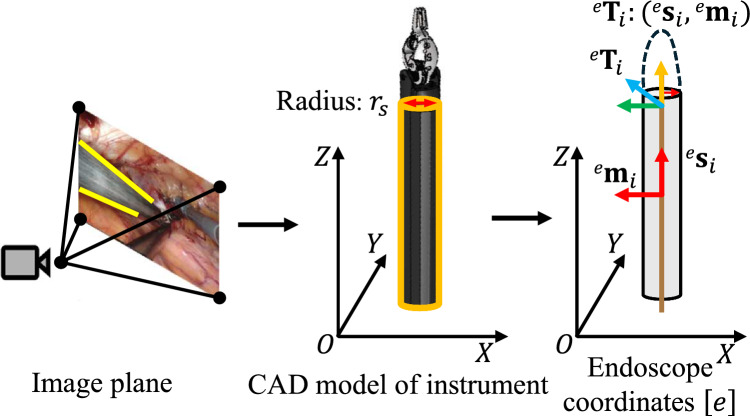
Geometric modeling of surgical instruments in endoscopic view

As shown in Fig. 2, most surgical instruments feature a cylindrical shaft with a constant radius $r_s$. Given an endoscopic frame *i*, we can compute the 3D pose ${}^e\textbf{T}_i \in \mathbb {R}^{3\times 4}$ of the tool based on its geometric primitives (i.e., boundaries and tip), where *e* represents the endoscope coordinates. The instrument’s axis at pose $^e\textbf{T}_i$ in the endoscope frame can be expressed using Plücker coordinates $\left( {}^e\textbf{s}_i, {}^e\textbf{m}_i\right)$, where ${}^e\textbf{s}_i \in \mathbb {R}^{3 \times 1}$ is a unit vector indicating the direction of the tool’s shaft, and ${}^e\textbf{m}_i \in \mathbb {R}^{3 \times 1}$ represents the moment of the tool. Let ${}^e\textbf{c}_j$ be a 3D point on the surface of the tool and denote its homogeneous coordinate as ${}^e\widetilde{\textbf{c}}_j = [{}^e\textbf{c}_j \; 1]^\textsf{T}$. Based on the geometric modeling of cylindrical objects [19], the following relationship can be established:

1 $$\begin{aligned} {}^e\widetilde{\textbf{c}}_j^\textsf{T} \cdot \begin{bmatrix} [{}^e\textbf{s}_i]_\times \,[{}^e\textbf{s}_i]^\textsf{T}_\times &  [{}^e\textbf{s}_i]_\times \,{}^e\textbf{m}_i\\ {}^e\textbf{m}_i^\textsf{T}\,[{}^e\textbf{s}_i]_\times ^\textsf{T} &  \Vert {}^e\textbf{m}_i\Vert ^2 - r_s^2 \end{bmatrix} \cdot {}^e\widetilde{\textbf{c}}_j = 0. \end{aligned}$$

According to perspective projection theory, the Plücker coordinates of the tool’s axis can be associated with the edge boundaries $\left( \textbf{l}_i^- \in \mathbb {R}^3, \textbf{l}_i^+ \in \mathbb {R}^3 \right)$ of the shaft in the image plane as follows:

2 $$\begin{aligned} \textbf{l}_i^- = \textbf{K}^{-\textsf{T}} \cdot \left( \textbf{I} - \alpha [{}^e\textbf{s}_i]_\times \right) {}^e\textbf{m}_i, \quad \textbf{l}_i^+ = \textbf{K}^{-\textsf{T}} \cdot \left( \textbf{I} + \alpha [{}^e\textbf{s}_i]_\times \right) {}^e\textbf{m}_i. \end{aligned}$$

where $\textbf{K}$ represents the camera’s intrinsic and $\alpha = \frac{r_s}{\sqrt{\Vert {}^e\textbf{m}_i\Vert ^2 - r_s^2}}$. Using the shaft mask predicted by the tool segmentor, we can compute the edge boundaries from the mask contour. The coordinates $\left( {}^e\textbf{s}_i, {}^e\textbf{m}_i\right)$ can then be derived by combining Eq. 2 with the boundary equations from the 2D mask. This allows us to obtain the 3D pose of the surgical instrument from the Plücker coordinates $[{}^e\textbf{s}_i,{}^e\textbf{m}_i]$.

Next, we calculate the 3D points on the surface of the shaft based on the determined pose. By integrating these points with the corresponding depth information from the estimated relative depth map described in Sect. 2.1, the transformation scale parameters that relate relative depth to real-world measurements is computed. First, we transform $[{}^e\textbf{s}_i,{}^e\textbf{m}_i]$ into the Plücker matrix $\textbf{L}_i$ to represent the axis:

3 $$\begin{aligned} \textbf{L}_i = \begin{bmatrix} [{}^e\textbf{m}_i ]_\times &  {}^e\textbf{s}_i \\ -{}^e\textbf{s}_i^\textsf{T} &  0 \end{bmatrix}. \end{aligned}$$

The projection of the tool’s axis onto the image plane is calculated as $\textbf{l}_s = \textbf{K}^{-\textsf{T}} \cdot {}^e\textbf{m}_i$. The shaft tip $\textbf{p}_{s0} = [u_{s0} \; v_{s0} \; 1]^\textsf{T}$ is first predicted from the endoscopic image, where $(u_{s0},v_{s0})$ denotes the pixel coordinates in the image. Subsequently, the corresponding 3D point $\textbf{c}_{s0}$ along the axis of the shaft is computed as follows:

4 $$\begin{aligned} \begin{aligned}&\textbf{w} = [\textbf{K} | \textbf{0}_{3 \times 1} ]\cdot \textbf{L}_i \cdot [\textbf{K}[0][0] \ 0 \ \textbf{K}[0][2]\!-\!u_{s0} \ 0 ]^\textsf{T}, \\&\rightarrow \ \textbf{c}_{s0} = [\frac{\textbf{w}[0]}{\textbf{w}[3]} \ \frac{\textbf{w}[1]}{\textbf{w}[3]} \ \frac{\textbf{w}[2]}{\textbf{w}[3]} ]. \end{aligned} \end{aligned}$$

Here, $\textbf{w}$ is a vector calculated from $\textbf{L}_i$ and the camera’s intrinsic parameters. So the depth of the point $\textbf{c}_{f0}$ on the shaft’s surface is derived. Following the process, we compute the depth values $\left( z(\textbf{c}_{f0}), \cdots , z(\textbf{c}_{fn})\right)$ of 3D points reprojected by the pixel $\left( \textbf{p}_{s0}, \cdots , \textbf{p}_{sn}\right)$ along $\textbf{l}_s$. Then, the relative depth values for $\left( \textbf{p}_{s0}, \cdots , \textbf{p}_{sn}\right)$ from the depth map $\textbf{D}_i$ is obtained, defined as $\left( \textbf{D}_i[\textbf{p}_{s0}], \cdots , \textbf{D}_i[\textbf{p}_{sn}]\right)$. Consequently, the transformation scale parameters between the relative depth and real-world measurements can be solved in closed form as a standard least-squares problem:

5 $$\begin{aligned} \left( \eta , \gamma \right) = \mathop {\arg \min }\limits _{\eta , \gamma }\sum _{k=1}^{n}{\left( \eta \times z(\textbf{c}_{fk}) + \gamma - \textbf{D}_i[\textbf{p}_{sk}] \right) }. \end{aligned}$$

Finally, as illustrated in Fig. 1, the absolute depth of the monocular endoscopic image is recovered using the parameters $\left( \eta , \gamma \right)$.

### Automatic tracking in endoscope for distance perception

This section presents our approach for tracking regions of interest (ROIs) in endoscopic image sequences to enable distance perception. The proposed algorithm is designed to address challenges such as occlusions, background noise, illumination variations, and tissue deformation while supporting the tracking of multiple ROIs without requiring pre-training. The framework operates in two key phases: initialization and tracking.

*Initialization phase.* The initialization phase leverages a segmentation foundation model and a vision-language model to identify ROIs. For our implementation, we employ the Segment Anything Model (SAM) [20] for segmentation and Claude as the vision-language model, though the framework is compatible with alternative models. SAM is a Vision Transformer (ViT)-based model trained on the large-scale SA-1B dataset, demonstrating robust zero-shot segmentation capabilities. The vision-language model, Claude, processes SAM-generated masks alongside predefined text prompts to localize the target ROI. Additionally, an interactive segmentation method is available, where surgeons manually select ROIs via clicks, prompting SAM to generate the corresponding mask.

*Tracking phase.* For tracking, we adopt the XMem model [21], which utilizes a unified feature memory inspired by the Atkinson–Shiffrin memory model to address long-term video segmentation challenges. Given an initial mask (obtained during initialization), XMem tracks the object and generates masks for subsequent frames.

*Implementation workflow.* The proposed system begins by ingesting real-time endoscopic image sequences. During initialization, the first suitable frame is selected as the reference, where ROIs are identified either (1) automatically via SAM-generated segmentation masks filtered by a vision-language model using text prompts, or (2) interactively through surgeon-guided clicks refined by SAM. The tracked ROIs are then propagated across subsequent frames using XMem’s memory-augmented segmentation pipeline, with all outputs forwarded to the downstream module for distance computation.

## Experimental procedures

### Evaluation details

The proposed method was evaluated in four main areas: scale-aware depth estimation, instrument pose estimation, ROI tracking, and distance calculation. For depth estimation, both quantitative results and qualitative comparisons of 2D depth maps and corresponding 3D reconstructions were presented to demonstrate performance. Instrument pose estimation accuracy was visualized using 3D renderings. In ROI tracking, we demonstrated automatic segmentation using text prompts with a vision-language model (VLM), and quantitatively evaluated VLM recognition failure rates and tracking accuracy. For distance calculation, experiments showcased the framework’s application in endoscopic procedures. This included monitoring the distance between the endoscope and surrounding anatomy for safety, as well as tracking instrument-to-tissue distances. Quantitative errors were reported for both use cases.

### Datasets

We evaluated our method on a dataset of robotic prostatectomy videos from 10 cases using the DaVinci system. From these, 99 monocular endoscopic video clips were extracted for training and testing. The instruments used are standard DaVinci tools with cylindrical shafts of fixed 4.5 mm radius. The dataset was split into 3,947 training frames, 1,189 validation frames, and 1,410 testing frames. Original frames at $1280 \times 1024$ resolution were resized to $640 \times 512$ for experiments. Ground-truth depth maps were generated using standard stereo matching [22]. Compared to earlier datasets, the testing data include new clips with continuous frames featuring soft tissue cutting, camera motions, tool occlusions, and illumination changes. Some clips involve short camera pose adjustments ($\sim$1 s), while others focus on longer instrument cutting sequences lasting 12–15 s (360–450 frames). For quantitative 3D pose evaluation, several clips were also collected from five tasks in the SurRoL surgical simulator [23]. Additionally, the Hamlyn dataset [24] was used for further testing. Following the EndoDepth protocol [16], the dataset was split into training and testing sets. Two clips with 736 frames were extracted from testing for scale-aware depth evaluation. In the first, the scale was calculated from the initial frame and applied to subsequent frames for depth accuracy. The second clip includes challenging instrument–tissue interactions for evaluation.

### Implementation and evaluation metrics

The relative depth estimation training followed Monodepth2[17], using ImageNet pre-training and fine-tuning on our medical datasets. For tool segmentation, a lightweight U-Net with a VGG11 backbone [25] was used, featuring five downsampling scales. The segmentation model was trained on a public dataset [26] and applied to our surgical data. To reduce domain gap effects, morphological operations refined the predicted tool boundaries.

Depth estimation was evaluated using six standard metrics [27]: absolute relative error (Abs Rel), squared relative error (Sq Rel), root mean squared error (RMSE), log-scale RMSE ($\text {RMSE}_{log}$), $\delta <1.25$ (percentage of pixels within 20% of ground truth), and mean absolute error (MAE). ROI tracking accuracy was measured by Dice score, Intersection over Union (IoU), and Precision [28], along with the success rate of automatic ROI recognition. For distance evaluation, ground-truth distance variations were derived from depth maps and ROI masks, and absolute and standard errors between predicted and ground-truth distances were calculated.

## Results

### Evaluation of scale-aware depth estimation

Quantitative depth comparison results on the in-house surgery data are presented in Table 1, with all results rescaled using the ground-truth median scaling method. In addition to standard depth metrics, the means and standard errors of the re-scaling factors are reported to demonstrate scale-awareness capability. The proposed depth model achieves the best performance in terms of up-to-scale accuracy across all metrics. Specifically, the state-of-the-art baseline method attains an MAE of 8.564 mm, an RMSE of 11.032 mm, and an accuracy of 76.9%. In contrast, the proposed method improves these results to an MAE of 6.347 mm, an RMSE of 8.637 mm, and an accuracy of 88.0%. Notably, our model also achieves near-perfect accuracy in absolute scale estimation, indicating that geometric modeling effectively enables fine scale-aware depth estimation.

**Table 1:** Quantitative comparisons for scale-aware depth estimation on in-house data

Method	Scale	$\text {Error} \downarrow$					$\text {Accuracy} \uparrow$
		Abs Rel	Sq Rel	RMSE	$\text {RMSE}_{log}$	MAE	$\delta \!<\!1.25$
EndoSfM[29]	NA	0.165	2.481	11.032	0.200	8.564	0.769
AF-SfMLearner[30]	NA	0.211	4.432	13.435	0.250	10.342	0.725
ManyDepth[31]	NA	0.165	2.489	11.691	0.204	9.608	0.742
Depth Anything[15]	NA	0.179	3.734	15.371	0.220	11.177	0.710
DPT[13]	NA	0.180	3.201	13.024	0.222	9.997	0.719
EndoDepth Stereo[16]	1.197±0.147	0.182	3.078	13.194	0.226	10.285	0.699
Ours	**0**.**959**±**0**.**043**	**0**.**110**	**1**.**388**	**8**.**637**	**0**.**148**	**6**.**347**	**0**.**880**

Sq Rel, RMSE, and MAE are in mm. The closer the scale is to 1, the better. The best results are indicated in **bold**

Further evaluation on the public Hamlyn dataset is shown in Tables 2 and 3. Table 2 reports results when scale factors are computed per frame with ground-truth median scaling. The proposed method outperforms the EndoDepth Stereo baseline [16] with consistently lower errors across all metrics (e.g., RMSE reduced from 9.778 mm to 9.608 mm, MAE from 5.526 mm to 5.374 mm) and a slight increase in accuracy (0.955 vs. 0.954). The estimated scale factors are close to the ideal value of 1, with a mean of 1.009 and a standard deviation of 0.018, indicating robust scale estimation. Table 3 applies a scale factor from the first frame to all subsequent frames, simulating a practical scenario of fixed scale during continuous depth estimation. Under this setting, the proposed method maintains performance comparable to EndoDepth Stereo, with minimal differences in error metrics and accuracy. The scale factor mean of 1.055 with a standard deviation of 0.016 further demonstrates stable scale estimation under real-world conditions.

**Table 2:** Quantitative comparisons for scale-aware depth estimation on Hamlyn data

Method	Scale	$\text {Error} \downarrow$					$\text {Accuracy} \uparrow$
		Abs Rel	Sq Rel	RMSE	$\text {RMSE}_{log}$	MAE	$\delta \!<\!1.25$
EndoDepth Stereo[16]	0.988±0.012	0.050	1.193	9.778	0.095	5.526	0.954
Ours	**1**.**009**±**0**.**018**	**0**.**048**	**1**.**150**	**9**.**608**	**0**.**093**	**5**.**374**	**0**.**955**

**Table 3:** Quantitative comparisons for scale-aware depth estimation on Hamlyn data

Method	Scale	$\text {Error} \downarrow$					$\text {Accuracy} \uparrow$
		Abs Rel	Sq Rel	RMSE	$\text {RMSE}_{log}$	MAE	$\delta \!<\!1.25$
EndoDepth Stereo[16]	1.004±0.008	0.055	0.940	7.659	0.108	3.940	0.949
Ours	1.055±0.016	0.056	0.944	7.679	0.108	4.030	0.950

For our method, the scale factor calculated from the first frame is applied to subsequent frames

**Fig. 3:**
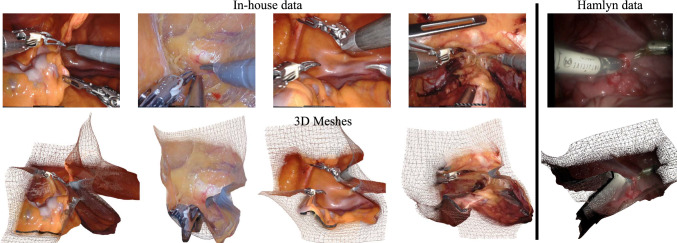
Typical examples of depth estimation results. The depth map generated by our scale-aware depth estimation model is converted into a 3D point cloud. This point cloud is then processed using mesh reconstruction techniques to create detailed 3D representations

Figure 3 illustrates our depth estimation results. The depth map generated by our framework is transformed into a 3D point cloud, which is then processed through mesh reconstruction techniques. This conversion allows for a comprehensive visual representation of the spatial relationships within the endoscopic scene. The resulting 3D point cloud effectively captures the complex structures of the surgical environment, including varying tissue depths and instrument placements. The mesh reconstruction further enhances this representation by creating a continuous surface that provides insights into the geometry of the scene. By analyzing the depth estimates, we can assess the accuracy and reliability of our framework in real surgical scenarios. The quality of the 3D reconstruction is pivotal for distance measurement, which is crucial for ensuring surgical precision and safety.

### Evaluation of instrument pose estimation

**Fig. 4:**
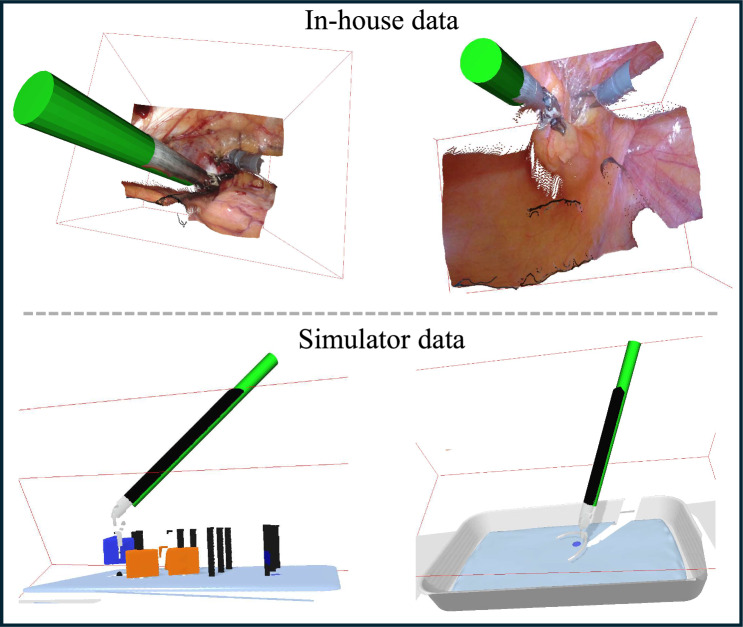
Qualitative comparison of 3D pose estimation. Green cylinders represent the rendered calculated poses of surgical tools

Quantitative evaluation on simulator data reveals average pose estimation errors of 1.459$^{\circ }$ and 3.220$^{\circ }$ in orientation, and 1.220 mm, 1.150 mm, and 2.356 mm in translation, demonstrating that the geometric modeling-based method achieves high accuracy in tool pose prediction. Additionally, qualitative comparisons, as shown in Fig. 4 with rendered surgical instrument poses alongside ground-truth depth maps, further confirm that the estimated 3D poses align well with ground-truth depth, validating the effectiveness of the proposed pose estimation approach.

**Fig. 5:**
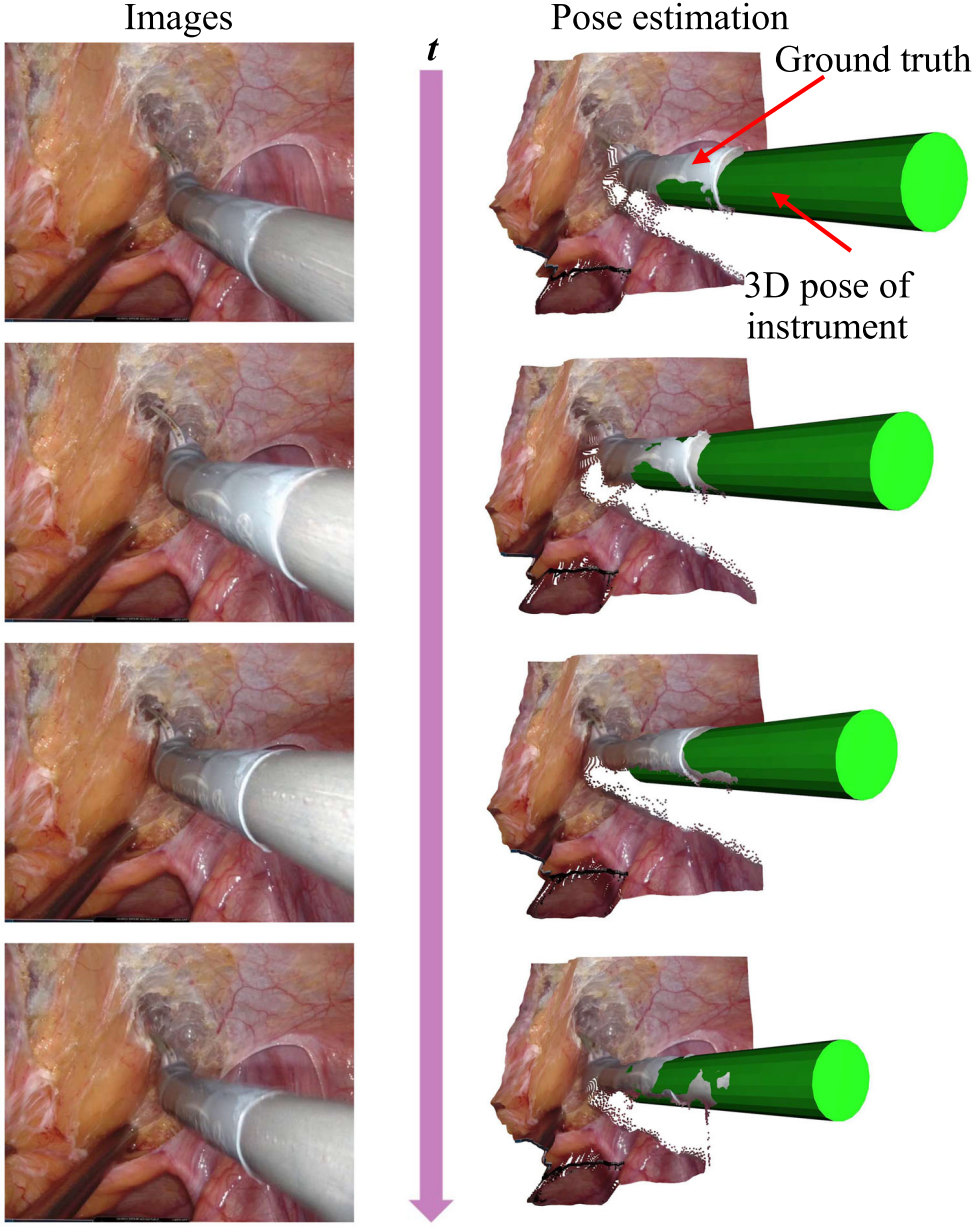
Instrument pose estimation results over time. Green cylinders represent the rendered calculated poses of surgical tools, illustrating their positions over continuous time

Figure 5 presents the instrument pose estimation results over continuous time. The calculated poses of the surgical instruments are rendered alongside ground-truth depth maps, providing a clear visual comparison between our estimates and actual measurements. By aligning the estimated poses with the ground-truth depth maps, we effectively evaluate the accuracy of our method in real-time scenarios. This visualization highlights the precision and robustness of our pose estimation framework in tracking the dynamic movements of surgical instruments during procedures.

### ROIs tracking

**Fig. 6:**
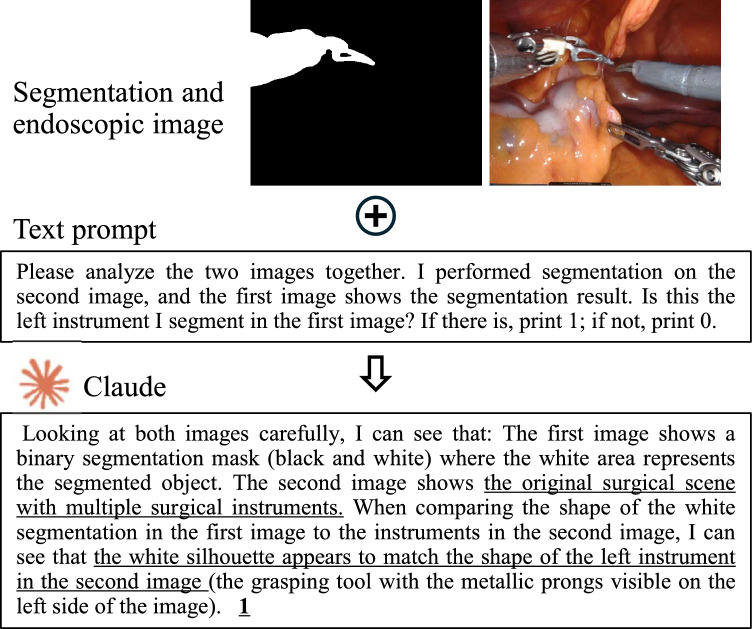
Automatic ROI recognition result using SAM and VLM

As shown in Fig. 6, automatic ROI detection for the initial frame is performed by inputting two images and a text prompt into Claude. The first image is a segmentation mask generated from the second image using SAM. The text prompt reads: “Please analyze the two images together. I performed segmentation on the second image, and the first image shows the segmentation result. Is this the left instrument I segmented in the first image? If there is, print 1; if not, print 0.” Claude analyzes the inputs and responds: “Looking at both images carefully, I can see that the first image shows a binary segmentation mask (black and white), where the white area represents the segmented object. The second image displays the original surgical scene with multiple surgical instruments. When comparing the shape of the white segmentation in the first image to the instruments in the second image, I observe that the white silhouette matches the shape of the left instrument in the second image (the grasping tool with the metallic prongs visible on the left side of the image). 1.” This result demonstrates that the VLM successfully identifies the left surgical instrument mask and shows a clear understanding of the surgical context and tool configurations.

The quantitative evaluation of the automatic tracking module is summarized in Table 4, covering recognition success rates and tracking accuracy metrics such as Dice score, IoU, and precision. Instrument recognition success reaches 75.86%, while tissue detection achieves 80.95%. Tracking performance is strong, with Dice scores of 95.24% for instruments and 92.92% for tissues, indicating high mask accuracy. IoU and precision consistently exceed 86% and 93%, respectively, demonstrating reliable and precise tracking during surgery. These results confirm the robustness and effectiveness of the pipeline in accurately detecting and tracking key ROIs, which is essential for downstream distance perception.

**Fig. 7:**
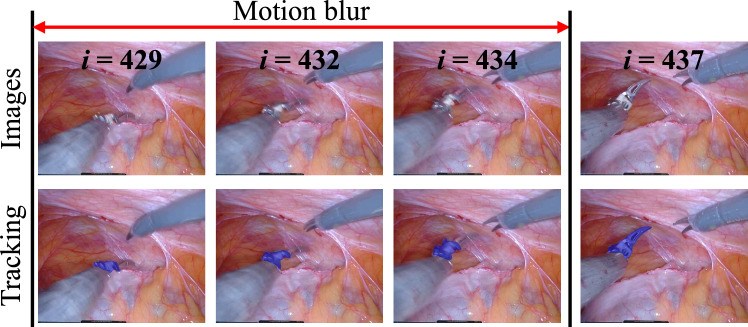
Failure cases. Rapid instrument movements within fractions of a second can cause the tracking model to struggle with precise localization. Variable *i* denotes the frame index in the video sequence

Despite the generally strong performance, some failure cases occur, as shown in Fig. 7. These failures mainly arise under challenging conditions such as motion blur caused by rapid instrument movements within fractions of a second. In such scenarios, the tracking model may struggle to precisely detect the correct instrument, resulting in tracking drift. Nevertheless, the model is still able to localize the instrument, albeit with reduced accuracy.

### Distance perception in endoscopic scenes

Distance perception in surgical scenes—encompassing tissue–instrument-endoscope distance and tissue–instrument distance—is crucial for achieving precision medicine and enhancing surgical safety. By integrating these three modules in Sect. 2, the proposed image-based framework enables robotic endoscopy with distance awareness, a critical component for ensuring the safety of automatic robotic surgery.

**Fig. 8:**
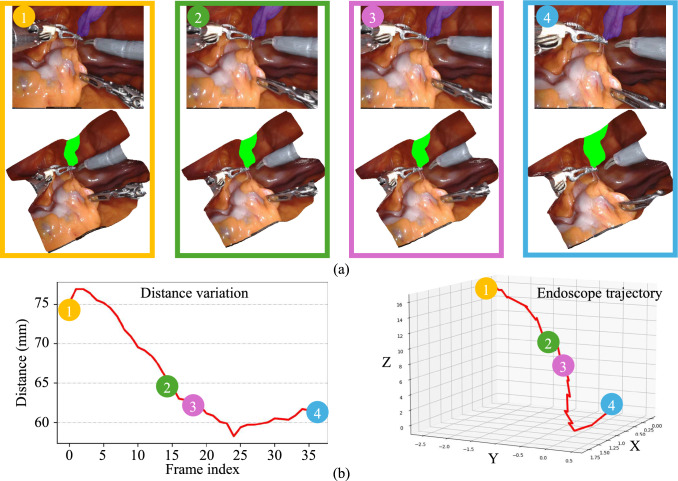
Distance variation from tissue to endoscope. **a** The first two rows display the endoscope image sequences, featuring tissue highlighted in transparent  alongside the reconstructed 3D point cloud, with tissue objects marked in . **b** The left graph illustrates the distance variation, while the right graph shows the corresponding camera trajectory [22]. Number circles (1, 2, 3, 4) correspond to specific frames and align with the matching numbered images in (**a**)

*Monitoring distance to endoscope.* Fig. 8 illustrates the distance from the tissue target within endoscopic views to the endoscope. The plotted ground-truth endoscope trajectory [22] illustrates the zoom-in motion of the endoscope, emphasizing how the distance to the tissue dynamically changes throughout the procedure. The distance variation graph reveals trends that closely align with the camera’s movements, reinforcing the system’s responsiveness to real-time alterations in the surgical environment. Moreover, tracking of the tissue ROI and the reconstruction of 3D structures derived from the depth maps are presented, demonstrating the high accuracy and stability of both the ROI tracking and depth estimation modules. Tissue targets are clearly marked on both the endoscopic image sequences and the 3D structures. These visualizations highlight that the tissue appears larger within the view as the endoscope zooms in, providing further evidence of the accuracy and reliability of our distance perception results.

**Fig. 9:**
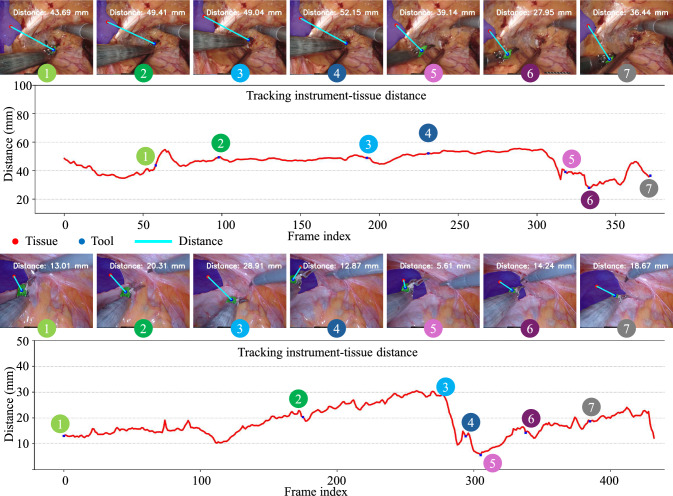
Tracking the distance between tools and tissue during robotic manipulation. Each example shows image sequences enhanced with tissue and instrument masks, along with distance measurements. The graph below illustrates the corresponding distance variations over time

*Tracking instrument-tissue distance.* As shown in Fig. 9, the robotic instruments are controlled to pull the tissue for a duration of 12 to 15 s. Throughout this process, the framework continuously calculates the distance between the tissue and the instrument tip. This involves tracking both objects even during occlusions and calculating their distance based on the depth estimation results. In the first row of each result, both the dynamic tissue and rigid instrument are localized accurately and stably over time. In the first dataset, the instrument initially moves away from the tissue, approaches it, and then moves away again, a motion clearly reflected in the accompanying image sequences. The distance variation graph below corroborates this observation, showing a corresponding trend. In the second dataset, a rapid approach of the instrument to the tissue occurs between frames 3 and 5. This quick movement is illustrated by the steep decline in the distance variation graph, indicating a significant reduction in distance over a short timeframe. Such responsiveness in the framework not only enhances the precision of surgical interventions but also reinforces the reliability of our distance measurement capabilities in real-time scenarios.

**Table 4:** Quantitative evaluation of the automatic tracking module

ROI	Automatic recognition	Tracking		
	Success rate $\uparrow$	Dice $\uparrow$	IOU $\uparrow$	Precision $\uparrow$
Instrument	75.86%	95.24	90.94	94.47
Tissue	80.95%	92.92	86.93	93.89

Table 5 summarizes the quantitative evaluation of the distance perception framework. The distance error is computed as the absolute difference between estimated distances and ground-truth measurements, reported as mean ± standard deviation in millimeters. The results indicate that the framework achieves an average distance error of approximately 4.7–6.4 mm across different measurement types, demonstrating reliable and precise distance estimation in complex surgical scenarios.

### Runtime

We also evaluated the runtime performance of the proposed framework. All source code was executed on Alienware desktop with an AMD Ryzen 9 7900 CPU and an NVIDIA RTX 4090 (24GB). Table 6 presents the average processing time per frame for endoscopic videos at a resolution of $320 \times 256$. The computational cost of the SAM or VLM is only incurred during the initialization phase of the ROI tracking module. Therefore, it is not included in the runtime performance evaluation. The overall distance perception model runs in real time at approximately 54 *ms* per frame ($\sim$19 fps), demonstrating its suitability for live surgical navigation.

### Ablation study

To study the effect of input resolution on the enhancement module, we performed a quantitative ablation on the relative depth estimation network. The results (Table 7) show that the multi-resolution fusion strategy significantly improves depth estimation quality. Additionally, using a high-resolution input of $480 \times 384$ achieves the best accuracy in our network.

## Discussion

Robotic systems have transformed minimally invasive surgery by improving surgeons’ precision and control. As automation advances, surgical robots need to perceive their environment autonomously, with distance awareness being essential, especially in endoscopic procedures with limited visual cues. We propose a monocular image-based framework for distance perception in surgical robots. It uses monocular endoscopic video and geometric constraints from the known cylindrical shape of instruments to recover absolute scale for scale-aware depth estimation. A tracking module also monitors tissue and instruments for accurate distance measurement. Evaluations on multiple clinical datasets show high accuracy in depth estimation, instrument pose, ROI tracking, and overall distance perception.

**Table 5:** Quantitative evaluation of our distance perception framework

Data		Distance error (*mm*)
Type	Im. #	
Tissue–camera	37	6.088±3.041
Tool–camera	33	5.637±4.208
Tissue–tool	373	6.355±2.976
	433	4.714±3.491

**Table 6:** Average runtime of the distance perception framework

Steps	Times (*ms*)
Depth estimation	21.6
Scale recovery	10.2
ROI tracking	22.3
Average	54.1

### Limitations

Despite encouraging results, several challenges remain. The tested datasets do not fully capture the variability of clinical cases, including diverse anatomies and pathologies. Motion blur from rapid movements and specular reflections on wet tissues significantly degrade image quality and depth estimation accuracy. While our enhancement module mitigates some artifacts, more robust solutions are needed for consistent performance across diverse clinical environments. Scale recovery relies on the known cylindrical geometry of instruments, limiting applicability to tools with well-defined shapes. Occlusions from tissue, blood, or overlapping instruments, as well as uncertainty in instrument radius measurements, reduce scale estimation accuracy. Additionally,

**Table 7:** Ablation studies of different input resolution in the enhancing module

High-res		Low-res	$\text {Error} \downarrow$			$\text {Accuracy} \uparrow$
$640 \times 512$	$480 \times 384$	$320 \times 256$	Abs Rel	RMSE	$\text {MAE}$	$\delta \!<\!1.25$
		$\checkmark$	0.113±0.045	9.008±3.038	6.524±2.068	0.878±0.073
$\checkmark$		$\checkmark$	0.113±0.045	8.881±3.028	6.424±2.068	0.881±0.072
	$\checkmark$	$\checkmark$	**0**.**110**±0.045	**8**.**698**±3.065	**6**.**322**±2.091	**0**.**886**±0.074

factors including segmentation uncertainty and imperfect boundary extraction can affect overall stability and accuracy. Future work could improve robustness by integrating additional scale cues such as illumination models [32] or multi-view constraints [33]. Although the tracking system incorporates advanced models for occlusion, bleeding, and dynamic changes, extreme cases with heavy occlusion or dense blood may still degrade performance. Our evaluation focused on relatively short sequences where scale remained stable. Over longer procedures, scale drift may occur due to accumulated errors. Future implementations will incorporate segment-wise processing and scale uncertainty estimation to trigger recalculation when uncertainty exceeds defined thresholds. Moreover, most evaluations relied on in-house data. Future work will include public datasets to better assess generalization and investigate how measured distances correlate with surgical risks to enhance safety.

### Feasibility of integration into flexible surgical robots

The proposed monocular endoscopic image-based distance perception framework is readily integrable into existing flexible surgical robotic systems (e.g., Agilis Robotics, Ltd. and Virtuoso Surgical, Inc.). Since flexible surgical platforms typically provide a monocular endoscopic video stream as standard, no hardware modifications are required. From a software perspective, the distance perception system will be developed as a modular component with clearly defined input/output interfaces, facilitating seamless embedding into robotic software architectures. To achieve real-time inference, crucial for intraoperative applications, we will optimize algorithm efficiency via GPU acceleration and streamlined model designs. Communication protocols such as ROS or platform-specific middleware will enable data exchange between the perception module and the robot’s control system, allowing downstream uses such as navigation, collision avoidance, or augmented reality overlays. Calibration routines will be incorporated to verify or update instrument geometry parameters (e.g., shaft radius), either via one-time setup or periodic intraoperative checks, ensuring sustained accuracy. Finally, surgeon-friendly visualization tools will be designed to overlay depth and distance information onto the endoscopic video feed, enhancing situational awareness without distraction.

### Future work

For clinical deployment, rigorous validation of safety, accuracy, and reliability is essential, including preclinical trials and human feasibility studies. Future work will extend validation to flexible surgical robots, which involve challenges like complex camera motion and tissue deformation. Animal and cadaver studies will help assess system robustness and clinical value. Integrating near-light illumination models [32] and multimodal data (e.g., fluorescence imaging) could improve absolute scale and depth accuracy. Advances in vision foundation models (e.g., Depth Anything [15], Depth Pro [34]) for efficient per-frame relative depth prediction [35, 36] offer promising improvements in accuracy, temporal consistency, and stability. For tracking, adding inter-frame pixel correspondences to estimate motion may reduce motion blur effects on instrument and tissue tracking. Finally, developing real-time intraoperative feedback to assist surgeons is a promising future direction.

## Conclusion

This work introduces a monocular image-based distance perception framework that uses geometric constraints from surgical instruments for scale-aware depth estimation and continuous tracking in endoscopic scenes. Looking forward, this technology has strong potential for integration into flexible surgical robots, forming a key foundation for enhanced autonomy and real-time safety monitoring. By delivering reliable spatial awareness, it can support advanced functions like autonomous tool navigation, collision avoidance, and intraoperative risk assessment, ultimately improving the safety and efficiency of robotic surgery.
